# Deoxynivalenol and its metabolite deepoxy-deoxynivalenol: multi-parameter analysis for the evaluation of cytotoxicity and cellular effects

**DOI:** 10.1007/s12550-016-0260-z

**Published:** 2016-11-05

**Authors:** Alexandra Springler, Sabine Hessenberger, Nicole Reisinger, Corinna Kern, Veronika Nagl, Gerd Schatzmayr, Elisabeth Mayer

**Affiliations:** 1 BIOMIN Research Center, Technopark 1, 3430 Tulln an der Donau, Austria; 2 Department of Applied Genetics and Cell Biology, University of Natural Resources and Life Sciences, Vienna, Austria

**Keywords:** Mycotoxin, IPEC-J2, Mitochondria, Apoptosis, MAPK signalling, In vitro

## Abstract

The mycotoxin deoxynivalenol (DON) contaminates agricultural commodities worldwide, posing health threats to humans and animals. Associated with DON are derivatives, such as deepoxy-deoxynivalenol (DOM-1), produced by enzymatic transformation of certain intestinal bacteria, which are naturally occurring or applied as feed additives. Using differentiated porcine intestinal epithelial cells (IPEC-J2), we provide the first multi-parameter comparative cytotoxicity analysis of DON and DOM-1, based on the parallel evaluation of lysosomal activity, total protein content, membrane integrity, mitochondrial metabolism and ATP synthesis. The study investigated the ability of DON and—for the first time of its metabolite DOM-1—to induce apoptosis, mitogen-activated protein kinase (MAPK) signalling, oxidative events and alterations of mitochondrial structure in porcine intestinal epithelial cells (IECs). The degree of DON toxicity strongly varied, depending on the cytotoxicity parameter evaluated. DON compromised viability according to the parameters of lysosomal activity, total protein content and membrane integrity, but increased viability according to assays based on mitochondrial metabolism and ATP synthesis. DON induced expression of cleaved caspase-3 (maximum induction 3.9-fold) and MAPK p38 and p42/p44 (maximum induction 2.51- and 2.30-fold, respectively). DON altered mitochondrial morphology, but did not increase intracellular ROS. DOM-1-treated IPEC-J2 remained unaffected at equimolar concentrations in all assays, thereby confirming the safety of feed additives using DON- to DOM-1-transforming bacteria. The study additionally highlights that an extensive multi-parameter analysis significantly contributes to the quality of in vitro data.

## Introduction

Mycotoxins are fungal secondary metabolites, which contaminate up to approximately 70% of all agricultural commodities (Streit et al. 2012). The fusariotoxin deoxynivalenol (DON) is one of the most prevalent of such food- and feed-associated contaminants (EFSA 2013; Rotter et al. 1996). Its toxicity depends primarily on species-specific susceptibility, as well as on severity and duration of exposure. Acute DON exposure in pigs can trigger diarrhoea, emesis, leucocytosis, haemorrhage, endotoxemia and, ultimately, shock-like death. Chronic low-dose exposure may result in growth retardation and immunological impairments (Pestka 2010a, b; Rotter et al. 1996). Especially pigs, whose diet is largely composed of cereal-rich feed, exhibit high DON susceptibility (Prelusky et al. 1994).

Associated with the occurrence of DON are also its derivatives, including those formed by fungi (3- and 15-acetyl-DON), plants (3-O-glucoside-DON), animals (DON-3- and DON-15-glucuronide and DON-sulphonates) or bacteria (deepoxy-deoxynivalenol; DOM-1) (Berthiller et al. 2013; Karlovsky 2011; Nagl and Schatzmayr 2015; Schwartz-Zimmermann et al. 2014). Polygastric animals and birds have high bacterial content located towards the beginning of the gastrointestinal tract. This allows at least partial conversion of DON to DOM-1 prior to reaching the small intestine, where absorption of the mycotoxin takes place (Maresca 2013). Nevertheless, relevance of DOM-1 in monogastric animals, where bacterial transformation of DON to DOM-1 is unlikely to occur prior to reaching the small intestine, arises due to the use of a feed additive, containing DON- to DOM-1-transforming bacteria (EC 2013a).

The cytotoxic nature of DON, as such, has been confirmed. It is widely assumed that, from a molecular point of view, DON toxicology is based on the interaction of its 12–13 epoxide moiety with the A-site of the eukaryotic 60S ribosomal subunit, generating so-called ribotoxic stress (Garreau de Loubresse et al. 2014; Pestka 2010a; Pierron et al. 2016). However, the numerous in vitro studies dealing with cytotoxic effects of DON report strikingly different cytotoxic potentials of this mycotoxin (Cheli et al. 2014; Gutleb et al. 2002). This is not only due to the use of diverse cells and cell lines but also results from the variety of in vitro assays available for evaluation of cytotoxicity. These include the neutral red (NR), the sulforhodamine B (SRB), the lactate dehydrogenase (LDH) and the colorimetric 3-(4,5 dimethylthiazol-2-yl)-2,5-diphenyltetrazolium bromide (MTT) assay, measuring lysosomal activity, total protein content, membrane integrity and metabolic activity, respectively. Nevertheless, despite their limitations, in vitro tests provide a reproducible microenvironment for assessing mycotoxin toxicity, modes of action and biological functions. They therefore present a suitable alternative or prerequisite to subsequent in vivo animal testing (Cheli et al. 2014). With direct regard to mycotoxins, it has been highlighted that the establishment of reliable in vitro toxicity data must be ensured by considering not only specificity and sensitivity of the cell line, culture conditions and the cell culture system itself but also the cellular endpoint parameters being measured (Gutleb et al. 2002). Considering that many studies are based on a single cytotoxicity parameter to identify a suitable non-cytotoxic DON concentration for implementation in further experiments, awareness of potential assay-dependent deviations of toxicity results is crucial.

Due to the oral route of DON exposure and the fact that IECs are confronted with high mycotoxin concentrations upon consumption of contaminated food or feed (Bouhet and Oswald 2005; Bracarense et al. 2012), the present study examines the effect of DON on viability and function of cells forming this organ system. In contrast to cancer cell lines, IPEC-J2 (Berschneider 1989) are neither transformed nor tumourigenic and exhibit strong morphological and functional resemblance of IECs in vivo (Schierack et al. 2006).

Thus, this study provides an analysis of the toxicological profile of DON and its metabolite DOM-1, thereby extending current knowledge regarding toxicological effects of both compounds and confirming the safety of DON- to DOM-1-transforming feed additives. It presents the first in vitro comparison of DON and its metabolite DOM-1, considering—in parallel—six different cytotoxicity parameters, which were further substantiated by investigation of more specific cellular events, such as apoptosis, MAPK signalling and impact on morphological integrity of mitochondria. Especially the comparative analysis of cytotoxicity highlights the considerable dependence of in vitro cytotoxicity data on the chosen assay.

## Materials and methods

### Cell culture

The porcine intestinal epithelial cells IPEC-J2 (ACC701; Leibniz Institute DSMZ (German Collection of Microorganisms and Cell Cultures, Braunschweig, Germany)) were routinely cultured as previously described (Springler et al. 2016). Cells were used up to passage 15. For cytotoxicity and oxidative stress assays, cells were seeded at 3 × 10^4^ in 96-well plates (Eppendorf, Hamburg, Germany). For total protein extraction, cells were seeded at 6 × 10^4^ cells/well in 12-well plates (Eppendorf, Hamburg, Germany). For immunoblotting and mitochondrial staining, cells were seeded in six-well plates (Eppendorf, Hamburg, Germany) or chamber slides (ibidi, Martinsried, Germany), respectively, at 1 × 10^5^ cells/well. For all assays, cells were allowed to differentiate for 8 days prior to treatment.

### Preparation of DON and DOM-1 stock solutions

DON (Biopure, Romer Labs®, Tulln, Austria) was dissolved in sterile distilled water to a concentration of 6.75 mM, which was further diluted to required concentrations in an assay-specific diluent. DOM-1 (Biopure, Romer Labs®, Tulln, Austria) was obtained as liquid calibrant solution (180.15 μM in acetonitrile). Required volumes were evaporated to dryness under nitrogen gas and re-dissolved in appropriate volumes of complete cultivation medium or Hank’s balanced salt solution (HBSS) (Sigma-Aldrich, St. Louis, MO, USA).

### Cytotoxicity

IPEC-J2 viability was assessed on the basis of lysosomal function (NR assay), total protein content (SRB assay), cell membrane integrity (LDH assay), metabolic activity (MTT and 1((4-[3-(4-iodophenyl)-2-(4-nitrophenyl)-2H-5-tetrazolio]-1,3-benzene disulfonate (WST-1) assay) and intracellular ATP concentration (CellTiter-Glo® (CTG) assay). Following differentiation, cells were treated with either complete cultivation medium (cell control), DON or DOM-1 (both 5–100 μM in complete cultivation medium) for 24, 48 and 72 h. All assays were performed in triplicates and in four independent experiments. Optical densities (ODs) or luminescence signals were measured using a microplate reader (BioTek Instruments Inc., Winooski, VT, USA).

#### NR, SRB and LDH assay

DON, DOM-1 and control treatments were removed, and the NR and SRB assays (both Aniara, West Chester, OH, USA) as well as the LDH assay (Pierce LDH Cytotoxicity Assay Kit, Thermo Scientific Inc., Waltham, MA, USA) were conducted according to the manufacturer’s specifications as previously described (Springler et al. 2016).

#### WST-1 and MTT assay

For the WST-1 assay (Roche, Rotkreuz, Switzerland), treatments were removed and cells were incubated with a 10% WST-1 solution in complete cultivation medium for 2 h at 39 °C. Formation of formazan dye was quantified at 450 nm, with a reference filter of 690 nm. For the MTT assay (Molecular Probes, Life Technologies, Carlsbad, CA, USA), treatments were removed and replaced by 100 μl HBSS. Subsequently, cells were incubated with 12 mM MTT for 4 h. Crystalline formazan was dissolved in 50 μl dimethyl sulfoxide (DMSO) (Sigma-Aldrich, St. Louis, MO, USA) for 10 min at 39 °C while shaking. OD was measured at 570 nm.

#### CellTiter-Glo® assay

Untreated and DON- or DOM-1-treated cells were equilibrated to room temperature for 30 min. According to the manufacturer’s instructions, CellTiter-Glo® Reagent (CellTiter-Glo® Luminescent Cell Viability Assay, Promega, Fitchburg, WI, USA) was added to each well in volumes equal to the amount of the cell culture medium. The plate was shaken for 2 min and subsequently incubated for 10 min at room temperature before luminescence was measured (integration time 1 s).

### Total protein extraction and quantification

Following differentiation, cells were incubated with complete cultivation medium (cell control) or DON (5–100 μM) in complete cultivation medium for 24, 48 and 72 h. For protein extraction, cells were washed twice with ice-cold phosphate-buffered saline (PBS) (Sigma-Aldrich, St. Louis, MO, USA), incubated with radioimmunoprecipitation assay (RIPA) buffer (Sigma-Aldrich, St. Louis, MO, USA) supplemented with 1× cOmplete™, Mini Protease Inhibitor Cocktail (Roche, Rotkreuz, Switzerland) for 5 min on ice, harvested by scraping and transferred to microcentrifuge tubes for 30 min on ice. Cells were centrifuged (10,000×*g*, 30 min, 4 °C) and supernatants collected. Protein concentration was determined via the bicinchoninic acid (BCA) assay (Thermo Fisher Scientific, Waltham, MA, USA) and a bovine serum albumin (BSA) standard curve.

### SDS-PAGE and immunoblotting

Immunoblot assays were performed to examine the expression of cleaved caspase-3 or MAPK activity. For cleaved caspase-3, cells were then treated with complete cultivation medium (cell control), DON (5–100 μM) or DOM-1 (100 μM), diluted in complete cultivation medium, for 24 h. For MAPK activity, complete cultivation medium was replaced by Dulbecco’s modified Eagle’s medium without supplements during the final 24 h of differentiation, to avoid high basal MAPK activation due to media components. Subsequently, cells were treated with Dulbecco’s modified Eagle’s medium (no supplements) (cell control), DON (10–30 μM) or DOM-1 (100 μM), diluted in Dulbecco’s modified Eagle’s medium (no supplements) for 1 h. Protein extraction and determination of protein concentration (BCA assay) was determined as described above.

Samples (20 μg) were loaded on sodium dodecyl sulphate (SDS) polyacrylamide gel in parallel with the prestained ladder (SM1811, 10–250 kD; Thermo Fisher Scientific, Waltham, MA, USA) and subsequently transferred onto a PVDF membrane by semi-dry electroblotting for 50 min. Membranes were blocked in 5% *w*/*v* skimmed milk power (Sigma-Aldrich, St. Louis, MO, USA), 1× Tris-buffered saline (TBS) and 0.1% Tween® 20 (Sigma-Aldrich, St. Louis, MO, USA) for 1.5 h at room temperature. For assessment of apoptosis, membranes were incubated with anti-rabbit cleaved caspase-3 (Asp175) (5A1E) rabbit monoclonal antibody (1:1,000; Cell Signalling, Danvers, MA) in 5% *w*/*v* BSA, 1× TBS and 0.1% Tween® 20 overnight at 4 °C with gentle shaking. For examination of MAPK activity, membranes were probed with rabbit anti-phospho-p44/42 ERK MAPK (1:1,000), rabbit anti-endogenous-p44/42 ERK MAPK (1:1,000), rabbit anti-phospho-p38 MAPK (Thr180/Tyr182) (3D7) and rabbit anti-endogenous-p38 (1:1,000) (all Cell Signalling, Danvers, MA) in 5% *w*/*v* BSA, 1× TBS and 0.1% Tween® 20 overnight (4 °C while gently shaking). In all experiments, detection of ß-actin with (13E5) rabbit monoclonal antibody (1:2,000; Cell Signalling, Danvers, MA) was used as internal loading control. Following incubation, membranes were washed and incubated with alkaline phosphatase-labelled goat anti-rabbit IgG (Sigma-Aldrich, St. Louis, MO, USA) for 1.5 h at room temperature with gentle shaking. After washing, blots were developed in substrate buffer (100 mM Tris, pH 9.5, 100 mM NaCl, 5 mM MgCl_2_) supplemented with 5-bromo-4-chloro-3-indolyl phosphate disodium salt (BCIP) and nitroblue tetrazolium chloride (NBT) (both Thermo Fisher Scientific, Waltham, MA, USA). Membranes were analysed using myImageAnalysis™ Software (Thermo Fisher Scientific, Waltham, MA, USA).

### Oxidative stress

#### Ratio reduced glutathione to oxidized glutathione

Total glutathione (GSH + GSSG) and oxidized glutathione (GSSG) of DON- and DOM-1 (both 5–100 μM)-treated cells (GSH/GSSG-Glo™ Assay, Promega, Fitchburg, WI, USA) were determined. For this, differentiated IPEC-J2 were treated with provided assay buffer (cell control), DON or DOM-1 (both 5–100 μM) or with positive control H_2_O_2_ (1 mM) (Sigma-Aldrich, St. Louis, MO, USA) (all diluted in provided assay buffer) for 45 min at 39 °C and 5% CO_2_. Subsequently, treatments were removed and replaced with either total glutathione lysis reagent or oxidized glutathione lysis reagent. Finally, luciferin lysis reagent was added to all wells for 30 min, followed by luciferin detection reagent for 15 min. Luminescence was read, and GSH/GSSG ratios were calculated directly from relative luminescence unit (RLU) measurements. The GSSG reaction signal was subtracted from that of the total glutathione signal to yield the value of reduced glutathione in the sample. For determining intracellular oxidative stress of DON and DOM-1, the 2′,7′-dichlorofluorescein diacetate (DCFH) (Sigma-Aldrich, St. Louis, MO, USA) assay was performed. Following differentiation, cells were washed with HBSS and subsequently exposed to 40 μM DCFH in HBSS for 1 h at 39 °C and 5% CO_2_. Cells were then washed with HBSS and treated with HBSS (cell control), DON (5–100 μM) or DOM-1 (100 μM). Fluorescence was measured after 1, 4, 6 and 24 h using excitation and emission wavelengths of 480 and 530 nm, respectively.

### Confocal laser scanning microscopy

Following differentiation, IPEC-J2 were treated with DON (30–100 μM) for 24 h and subsequently incubated with 25 nM MitoTracker Deep Red 633 (Life Technologies, Carlsbad, CA, USA) for 30 min, fixed with 3.7% formaldehyde in PBS and counterstained with 150 nM 4′,6-diamidino-2-phenylindole (DAPI) (Life Technologies, Carlsbad, CA, USA) in PBS. Images were captured using a laser scanning confocal microscope (Leica SP5 II, Wetzlar, Germany).

### Statistics

Statistical analysis was performed with IBM® SPSS Statistics 19.0 (SPSS Inc., Chicago, IL, USA). Values of each independent experiment were expressed as means of triplicates ± standard deviation (SD). All values were analysed for normality (Shapiro-Wilk) as well as homogeneity of variance (Levene statistics). Normally distributed homogenous data were analysed by analysis of variance (ANOVA) and the Dunnett’s *t* test compared to the control (DON: NR, SRB data after 24 and 48 h, LDH, WST, MTT data after 24 and 48 h, CTG data after 24 and 72 h; GSH/GSSG; DCFH data after 24 h; apoptosis; DOM-1: all cytotoxicity assays, GSH/GSSG, DCFH data after 24 h, apoptosis). If data were normally distributed but not homogenous, ANOVA and the Dunnett’s T3 test (DON: MTT data after 72 h, CTG data after 48 h, SRB after 72 h, DCFH data after 1, 4 and 6 h; DOM-1: DCFH data after 1, 4 and 6 h) or the Tamhane T2 test (DON and DOM-1: p38 and p44/42 activation) was used. If normal distribution was violated, the Kruskal-Wallis test was used (BCA). Significances were marked with asterisks (*p < 0.05, **p < 0.01 and p*** < 0.001). IC50 values were calculated using GraphPad Prism 5.0 (GraphPad Software, Inc. La Jolla, CA, USA).

## Results

### Lysosomal activity (NR assay)

**Fig. 1:**
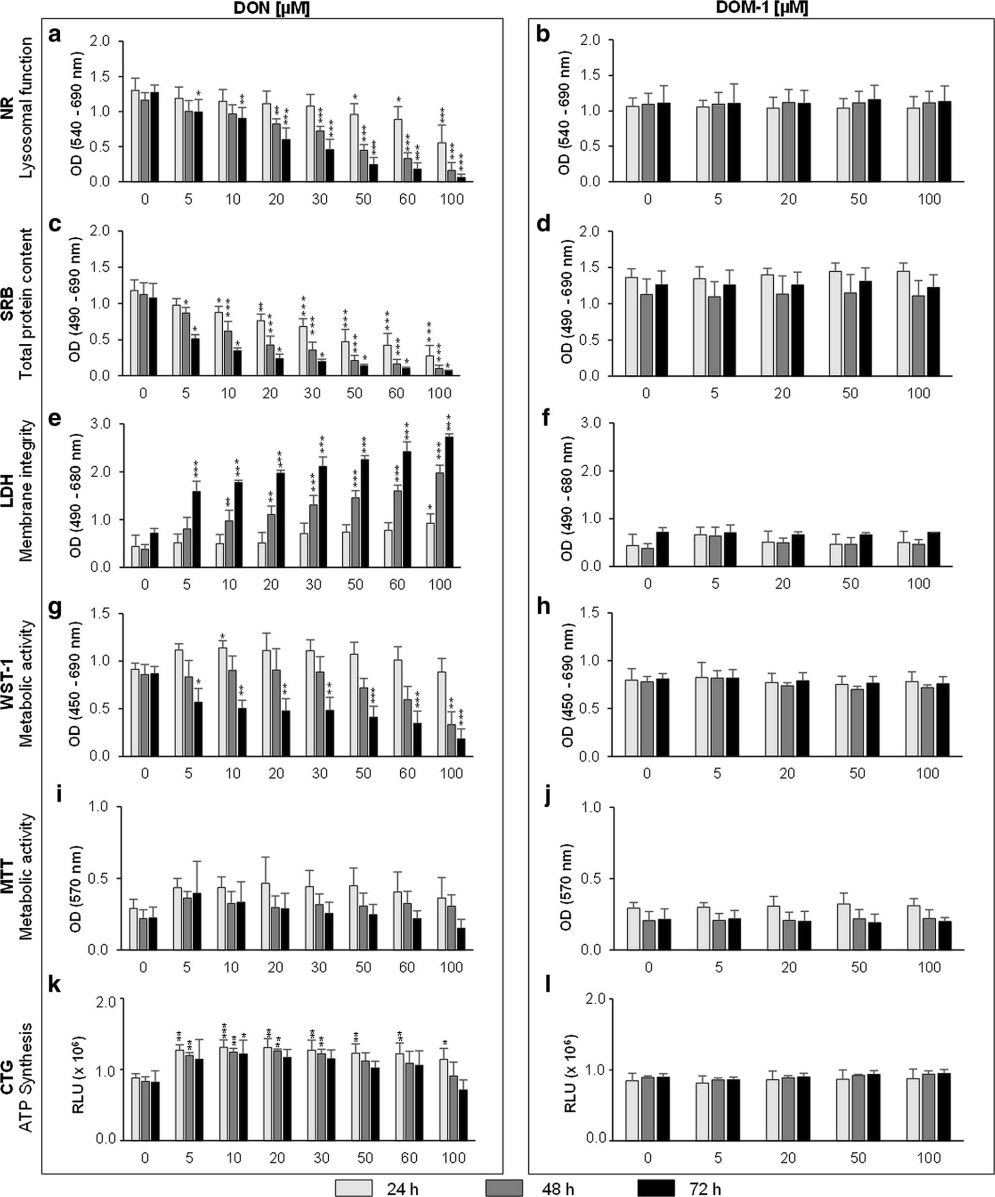
DON differentially affected viability of IPEC-J2 while DOM-1-treated cells remained unaffected. Differentiated IPEC-J2 were treated with DON or DOM-1 (5–100 μM) for 24, 48 and 72 h. Cytotoxicity was evaluated based on lysosomal function (NR) (**a**, **b**), total protein content (SRB) (**c**, **d**), membrane integrity (LDH) (**e**, **f**), metabolic activity of mitochondria (WST-1 (**g**, **h**) and MTT (**i**, **j**)) and ATP synthesis (CTG) (**k**, **l**). Data represent mean ± SD, *n* = 4. The dataset was analysed via the Dunnett’s *t* test or the Dunnett’s T3 test (for details see the “Materials and methods” section). *Asterisks* indicate significant difference compared to control of the respective time point (*p < 0.05; **p < 0.01; and ***p < 0.001)

Compared to the control at respective time points, 100 μM DON reduced viability by a maximum of 58% (*p* < 0.001) after 24 h, 86% (*p* < 0.001) after 48 h and 95% (*p* < 0.001) after 72 h (Fig. 1a). IC50 values were determined after 48 h (IC50 44.83 ± 6.14 μM) and 72 h (IC50 25.93 ± 5.13 μM) of DON treatment. DOM-1 did not negatively affect viability of IPEC-J2 at any tested concentration (Fig. 1b).

### Total protein content (SRB and BCA assay)

**Fig. 2:**
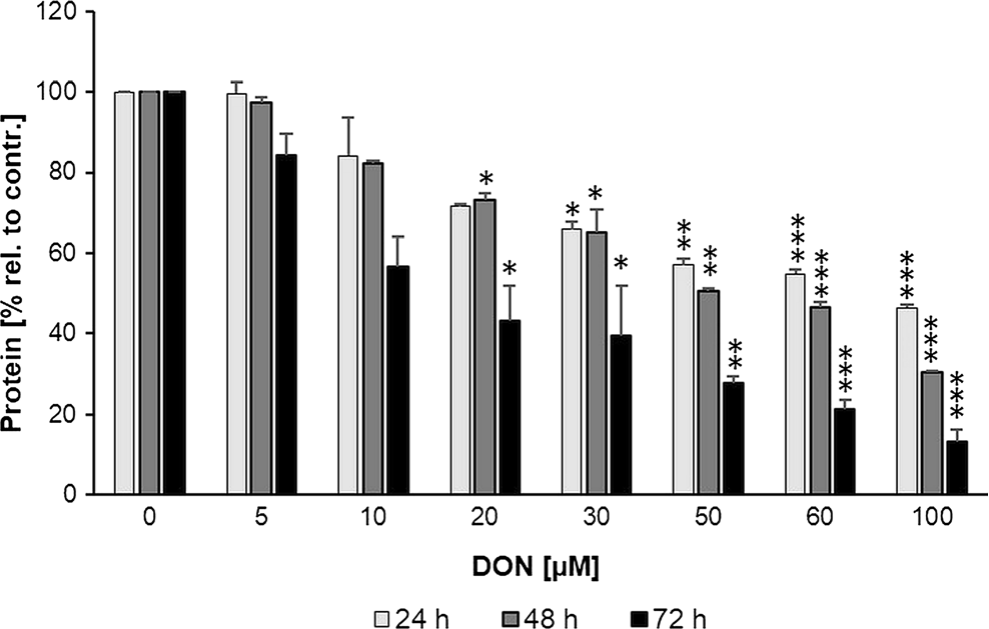
DON reduced total protein content. Differentiated IPEC-J2 were treated with DON (5–100 μM) for 24, 48 and 72 h. Total protein was extracted and quantified via the BCA assay. Data was normalized to control and represent mean ± SD, *n* = 4. The dataset was analysed via the Kruskal-Wallis test (for details see the “Materials and methods” section). *Asterisks* indicate significant difference compared to control of the respective time point (*p < 0.05; **p < 0.01; and ***p < 0.001)

Compared to the control at respective time points, 100 μM DON decreased viability by a maximum of 77% (*p* < 0.001) after 24 h, 91% (*p* < 0.001) after 48 h and 97% (*p* = 0.017) after 72 h (Fig. 1c). IC50 values were determined after 24 h (IC50 35.97 ± 11.85 μM), 48 h (IC50 18.90 ± 5.70 μM) and 72 h (IC50 2.47 ± 1.94 μM). Equimolar amounts of DOM-1 (5–100 μM) did not reduce IPEC-J2 viability (Fig. 1d). According to the BCA assay, 100 μM DON decreased protein content by approximately 54% (24 h), 70% (48 h) and 87% (72 h) (Fig. 2).

### Membrane integrity (LDH assay)

No significant LDH release was detected after the 24-h DON (5–60 μM) treatment. Only at 100 μM were LDH levels significantly higher than that of the control, reaching 0.93 ± 0.20 (*p* = 0.015) compared to the control (0.44 ± 0.24) (+111%). After 48 h, significant LDH increases were observed at 10–100 μM and after 72 h between 5 and 100 μM DON (Fig. 1e). DOM-1 did not induce LDH release exceeding that of the control at any tested concentration (Fig. 1f).

### Metabolic activity (WST-1 and MTT assay)

According to the WST-1 assay, DON did not decrease viability at any test concentration after 24 h. At 10 μM DON, viability was even significantly increased by approximately 25% (*p* = 0.049) compared to the control. At 100 μM, viability reached control levels. After 48 h, OD was approximately equal to the control between 5 and 30 μM and below the control at higher concentrations. At 100 μM, viability was significantly decreased by 61% (*p* = 0.001) compared to the control. Only after 72 h, DON (5–100 μM) significantly reduced viability, with a maximum reduction of 79% (*p* < 0.001) at 100 μM (Fig. 1g).

The MTT assay showed no significant DON-induced viability reduction; however, particularly high standard deviations were observed. Viability did not fall below that of the control at any DON concentration after 24 and 48 h. Only at 100 μM was DON viability below that of the respective control at 72 h (Fig. 1i). In contrast to the effects induced by DON, DOM-1 did not affect cell viability at any test concentration (Fig. 1h, j).

### Intracellular ATP (CTG assay)

According to the CTG assay, luminescence significantly exceeded control levels at all test concentrations after 24 h and between 5 and 30 μM after 48 h. Luminescence reached maxima at 10 μM (+49%, *p* < 0.001) and at 20 μM (+51.8%, *p* = 0.002), respectively. After 72 h, luminescence was significantly higher than that of the cell control only at 10 μM (+48%, *p* = 0.023). At remaining test concentrations, no statistically significant effect was observed. However, viability was above that of untreated control cells up to 60 μM DON (Fig. 1k). DOM-1 had no effect on cellular metabolism or intracellular ATP (Fig. 1l).

### Apoptosis

**Fig. 3:**
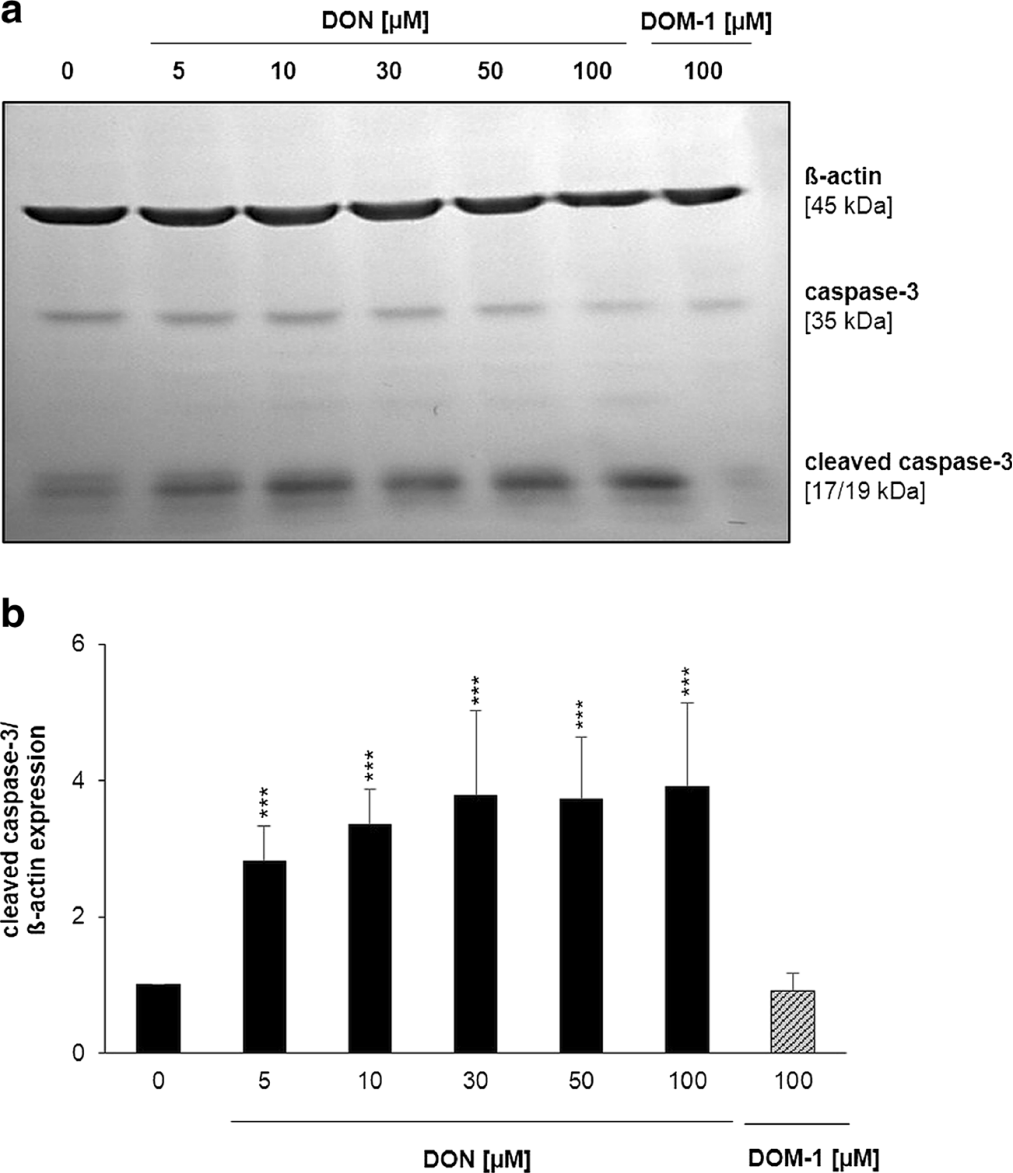
DON, but not DOM-1, induced caspase-3 activation. Differentiated IPEC-J2 were treated with DON (5–100 μM) or DOM-1 (100 μM) for 24 h. The expression of apoptosis indicator cleaved caspase-3 was analysed by immunoblotting (**a**) and by densitometry after normalization with ß-actin signals (**b**). Data was normalized to control and represent mean ± SD, *n* = 4. The dataset was analysed via Dunnet’s test (for details, see the “Materials and methods” section). *Asterisks* indicate significant difference compared to control (***p < 0.001)

DON significantly induced caspase-3 activation by a factor of 2.82 ± 0.53 at 5 μM (*p* < 0.001) and 3.36 ± 0.51 at 10 μM (*p* < 0.001) compared to the control. Between 30 and 100 μM DON, expression of cleaved caspase-3 was increased by a factor of 3.7–3.9 compared to the control (*p* < 0.001 for all DON concentrations). In comparison, DOM-1 (100 μM) did not induce expression of active cleaved caspase-3 in differentiated IPEC-J2. The protein expression of the housekeeping protein ß-actin remained stable under all concentrations (Fig. 3a, b).

### Effect of DON and DOM-1 on phosphorylation status of MAPK protein p38 and p44/p42

**Fig. 4:**
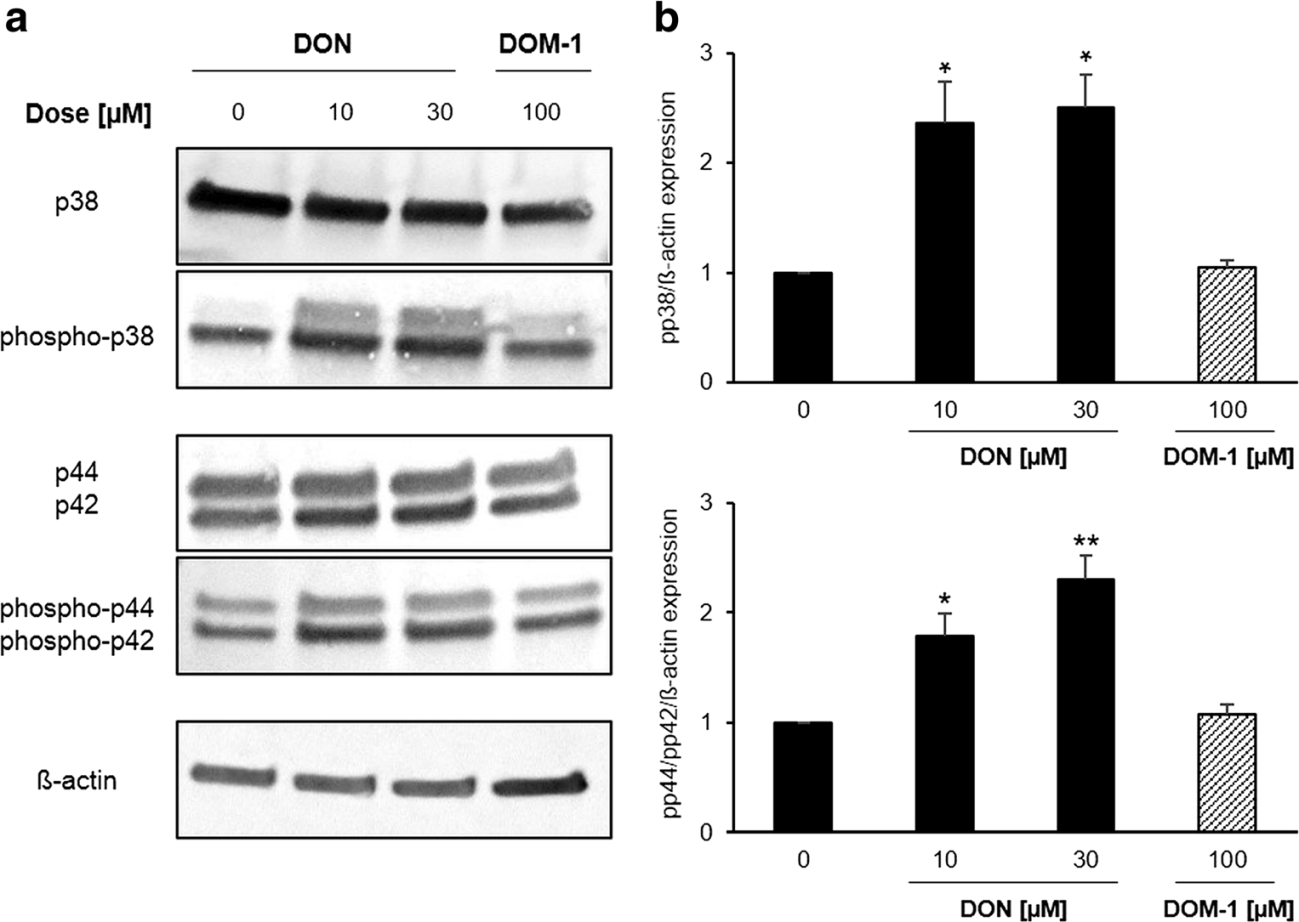
DON, but not DOM-1, induced MAPK protein p38 and p44/p42 activation. Differentiated IPEC-J2 were treated with DON (10–30 μM) or alternatively DOM-1 (100 μM) for 1 h. The phosphorylation status of p44/p42 and p38 was determined by immunoblotting (**a**) and by densitometry after normalization with endogenous signals and ß-actin (**b**). Data was normalized to control and represent mean ± SD, *n* = 4. The dataset was analysed via the Tamhane T2 test (for details, see the “Materials and methods” section). *Asterisks* indicate significant difference compared to control (*p < 0.05; **p < 0.01)

While DOM-1 (10–30 μM) did not affect the phosphorylation status of p38 or p44/p42, DON treatment (10–30 μM) indirectly induced phosphorylation of p38 and p44/42. At 10 and 30 μM, phosphorylated p38 was increased by a factor of 2.37 ± 0.37 (*p* = 0.031) and 2.51 ± 0.29 (*p* = 0.011), respectively. DON increased levels of phosphorylated p44/42 (ERK1/2) at 10 μM (1.79 ± 0.19 (*p* = 0.024)) and 30 μM (2.30 ± 0.22 (*p* = 0.008)). The expression of the housekeeping protein ß-actin remained stable under all conditions (Fig. 4a, b).

### Effect of DON and DOM-1 on oxidation level of differentiated IPEC-J2

**Fig. 5:**
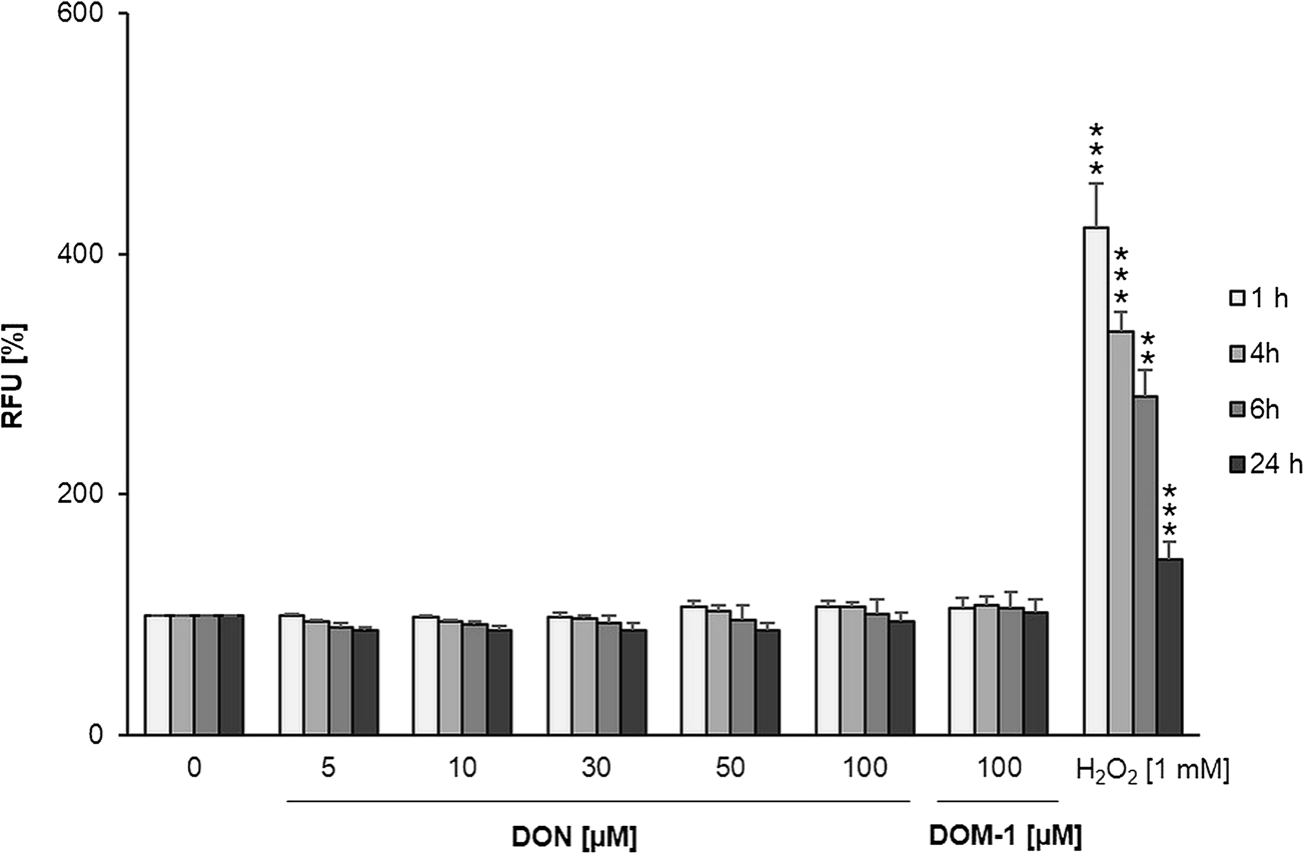
Neither DON, nor DOM-1, increased intracellular ROS concentrations. Differentiated IPEC-J2 were exposed to 2′,7′-dichlorofluorescein diacetate (DCFH) (40 μM) for 1 h and subsequently treated with DON (5–100 μM), DOM-1 (100 μM) or positive control H_2_O_2_ (1 mM) for 1, 4, 6 and 24 h. Data is presented as relative fluorescence units (RFU) and was normalized to negative control (untreated cells). The dataset was analysed via Dunnett’s *t* test or Dunnett’s T3 test (for details, see the “Materials and methods” section). Data represent mean ± SD, *n* = 4. *Asterisks* indicate significant difference compared to control (**p < 0.01 and ***p < 0.001)

Although the GSH/GSSG ratio was slightly below (−14% at 100 μM) the control, no statistically significant DON-induced reduction of the GSH/GSSG ratio was observed. In contrast, 1 mM H_2_O_2_ (positive control) decreased the GSH/GSSG ratio significantly by 43%, from 31.00 in untreated control cells to 17.52 (*p* = 0.001) (Table 1). DOM-1 did not decrease the GSH/GSSG ratio at any concentration. At 5 μM DOM-1, the GSH/GSSG ratio equalled 42.2 compared to the control (32.80). At 50 and 100 μM DOM-1, the ratio of GSH to GSSG was approximately at the level of control cells (Table 1). According to the DCFH assay, neither DON (5–100 μM) nor DOM-1 (100 μM) led to significant reactive oxygen species (ROS) generation over 24 h (Fig. 5).

**Table 1:** Neither DON, nor DOM-1, altered ratio of oxidized to reduced glutathione (GSH/GSSG)

	Total glutathione (GSH + GSSG)		Oxidized glutathione (GSSG)		Reduced/oxidized glutathione (GSH/GSSG)	
mM	H_2_O_2_		H_2_O_2_		H_2_O_2_	
1	1.97 × 10^6^ ± 1.76 × 10^4^		2.02 × 10^5^ ± 6.88 × 10^3^		17.52 ± 0.54* (*p* = 0.001)	
μM	DON	DOM-1	DON	DOM-1	DON	DOM-1
0	1.7 × 10^6^ ± 1.9 × 10^5^	1.7 × 10^6^ ± 2.3 × 10^5^	1.1 × 10^5^ ± 1.7 × 10^4^	9.9 × 10^4^ ± 1.1 × 10^4^	31.0 ± 5.6	32.8 ± 5.3
5	1.8 × 10^6^ ± 2. 0 × 10^5^	1.4 × 10^6^ ± 7.8 × 10^4^	1.2 × 10^5^ ± 2.5 × 10^4^	6.3 × 10^4^ ± 6.4 × 10^3^	27.3 ± 5.0	42.2 ± 7.4
50	1.8 × 10^6^ ± 1.8 × 10^5^	1.5 × 10^6^ ± 8.0 × 10^4^	1.3 × 10^5^ ± 2.7 × 10^5^	8.1 × 10^5^ ± 1.3 × 10^4^	27.0 ± 5.2	34.5 ± 3.9
100	1.8 × 10^6^ ± 9.3 × 10^5^	1.5 × 10^6^ ± 1.0 × 10^5^	1.3 × 10^5^ ± 2.9 × 10^5^	8.5 × 10^4^ ± 9.9 × 10^3^	27.2 ± 6.7	34.0 ± 4.5

Differentiated IPEC-J2 were treated with DON (5–100 μM) (*n* = 4), DOM-1 (5–100 μM) or positive control H_2_O_2_ (1 mM) (both *n* = 3) for 45 min. Total and oxidized glutathione were determined via the GSH/GSSG-Glo™ assay. The dataset was analysed via Dunnett’s *t* test (for details, see the “Materials and methods” section). Data represent mean ± SD. Asterisks indicate significant difference compared to control

*p < 0.01

### Effect of DON on mitochondrial morphology

**Fig. 6:**
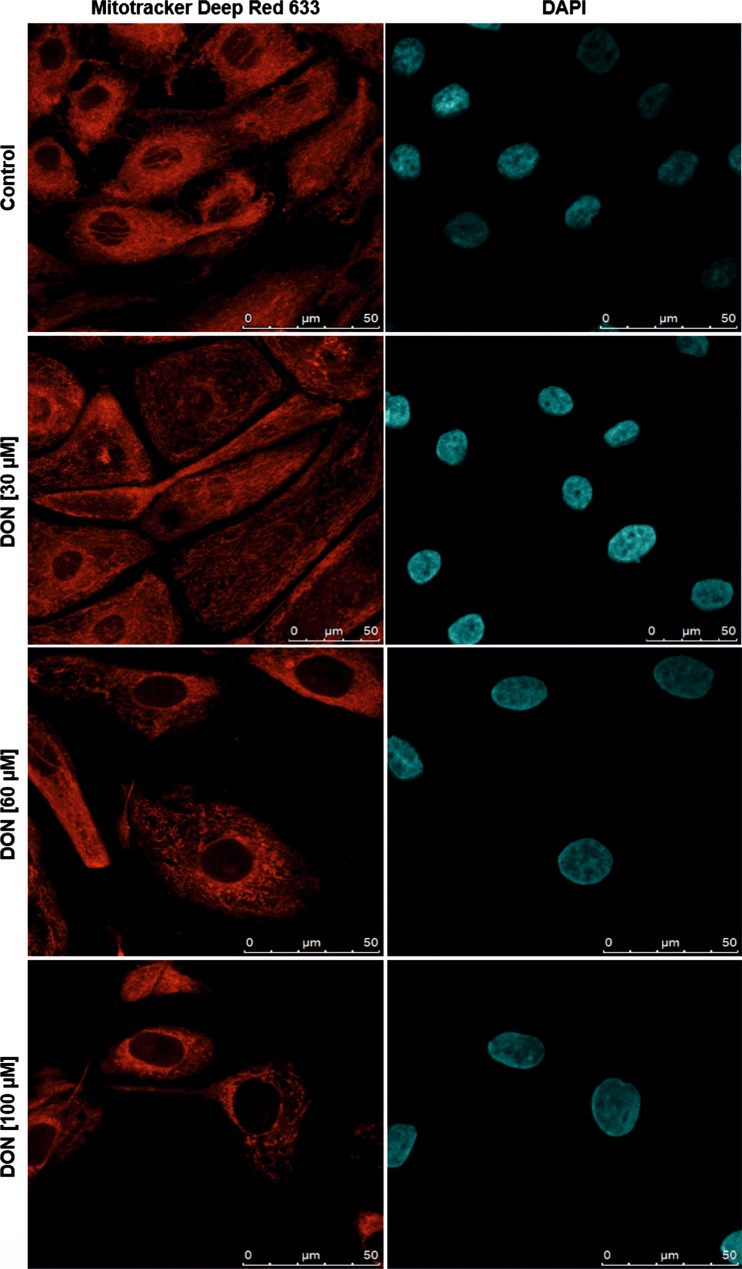
DON structurally altered the filamentous mitochondrial network and promotes granularization in vicinity of the nucleus. Differentiated IPEC-J2 were left untreated (control) or were exposed to DON (30–100 μM) for 24 h. Subsequently, cells were stained with MitoTracker Deep Red FM (25 nM), fixed with formaldehyde and counterstained with 4′,6-diamidino-2-phenylindole (DAPI) (150 nM). *Scale bar* 50 μm

Mitochondria in control cells stained with the fluorophore MitoTracker® Deep Red FM revealed thin, filamentous structures under a confocal laser microscope, similar to those previously reported in literature (Johnson et al. 1980; Karbowski et al. 1999b; Poot et al. 1996). However, mitochondria of cells treated with DON for 24 h led to a varied population of filamentous and granular structures. With increasing DON concentrations, mitochondria became predominantly granular, thicker and more condensed and located mainly in the vicinity of the nucleus. Furthermore, a slight increase in the nucleus size is observed with increasing concentrations of DON (Fig. 6).

## Discussion

In vitro cytotoxicity assays facilitate the detection of many mycotoxins, which potentially impair the biochemical activity of animal or human cells. The present study provides the first comparative in vitro analysis of DON and DOM-1 in porcine IECs, based on six cytotoxicity parameters. While we were able to confirm previous reports regarding the lack of DOM-1 toxicity, we demonstrated substantial variations with regard to DON cytotoxicity, depending on the employed assay type. Many existing in vitro DON studies are restricted to a single cytotoxicity assay, such as the MTT (Alassane-Kpembi et al. 2015; Wan et al. 2013), LDH (Diesing et al. 2011a; Pinton et al. 2009) or NR (Devreese et al. 2013; Vandenbroucke et al. 2011) assay. Their comparison reveals remarkable differences with respect to the degree of cytotoxicity, even for identical cell lines. In agreement with Cheli et al. (2014), we herein demonstrate that the inclusion of several toxicity-associated endpoints greatly improves the predictive power of in vitro tests, not just for mycotoxins, but for toxicants in general.

The presented in vitro analysis included DON concentrations between 5 and 100 μM. For the applied cell culture model, which does not discriminate between apical and basolateral compartments, the range of 5–30 μM DON, equivalent to food and feed contaminations of 1.5 to 10 mg/kg (Sergent et al. 2006), is particularly relevant. This not only corresponds to concentrations present in feed (Abouzied et al. 1991; Trucksess et al. 1995); it is also a concentration range which, when applied apically, negatively affects parameters such as intestinal barrier integrity (Pinton et al. 2009, 2010; Springler et al. 2016). In the case of basolateral exposition of IECs to DON, resembling in vivo contact to DON via the bloodstream, relevant concentrations are expected to be far lower (Danicke et al. 2004).

Strongest DON toxicity was reflected by the SRB, followed by the NR and LDH assays. This is particularly well illustrated after 24 and 48 h, where sensitivities to the toxin varied greatly between the three assays. Although we are the first to study DON-induced toxicity via SRB incorporation, Kluess et al. (2015) reported a 25% reduction of total protein content via western blotting in differentiated IPEC-J2 treated with 6.75 μM DON for 72 h. In contrast, we report reductions of 50 and 70% (SRB) and 16 and 43% (BCA) at 5 and 10 μM DON after 72 h, respectively. Nevertheless, the severe effect is in accordance with the primary toxicological feature of DON, namely its impairment of protein synthesis (Van de Walle et al. 2010). Although neither the SRB nor the BCA assay monitor de novo protein synthesis, the observed effect on protein content may—at least partially—be a downstream effect of DON-induced impairment of protein synthesis. With regard to the NR assay, a study on 21-day differentiated IPEC-J2 showed that 5 μg/ml (∼17 μM) and 10 μg/ml (∼33 μM) DON, when applied for 24 h, decreased IPEC-J2 viability by ∼15 and 20%, respectively (Vandenbroucke et al. 2011). Our analysis revealed comparable viability reductions of 15% at 20 μM and 18% at 30 μM DON.

Compared to the SRB and NR assay, the LDH assay showed far less severe DON toxicity. LDH leakage is not strictly a result of membrane damaging effects but can be a downstream event of apoptosis and subsequent secondary necrosis, where cytosolic components, such as LDH, leak to the cell exterior. Thus, detection of LDH in the supernatant does not permit discrimination between primary necrosis and secondary necrosis as a consequence of apoptotic cell death. The induction of cleaved caspase-3 and the absence of LDH in cell culture supernatant observed after 24 h however suggest that the observed LDH release after 48 and 72 h was a result of secondary necrosis of apoptotic cells. It should be noted that the faint band, indicating cleaved caspase-3 expression, in control cells most likely results from the presence of fetal bovine serum in the cultivation medium. Furthermore, we recognize the lack of a positive control (e.g. staurosporine) for detection of apoptosis.

The observed DON-induced apoptosis is likely to be a downstream effect of early p38 activation (Wu et al. 2014), which we detected after 1 h. DON most likely induced signalling cascades, which then led to p38 phosphorylation, a stress response to DON. A similar finding was made in macrophages, were p38 activation was detected at high DON doses (μM) (Pestka 2010a, c). The observed p44/p42 activation in DON-treated cells may be interpreted as a general cellular response to DON, possibly linked to the activation of cellular repair mechanisms. Additionally, MAPK p44/42 is involved in IEC morphology and tight junction signalling, which are negatively affected by DON (Pinton et al. 2009). DON-induced p44/42 activation has also been reported in porcine IECs of the ileum and jejunum (IPEC-1) (Pinton et al. 2010), mouse spleen (Zhou et al. 2003) and porcine dendritic cells (Sergent et al. 2006).

In contrast to the SRB, NR and LDH assays, the MTT, WST-1 and CTG assays delivered unusually high viability signals, often at the level of or exceeding the cell control. Despite limitations (lack of sensitivity, chemical interferences) and toxicity of the MTT compound (Riss et al. 2004), the MTT assay is commonly used to determine DON toxicity. While we found no DON-induced viability reductions via the MTT assay, Wan et al. (2013) reported viability reductions to 85 and 58% after 48 h in differentiated IPEC-J2 treated with 1 or 2 μM DON, respectively. Others found significant viability reductions of differentiated IPEC-J2 at 1000–4000 ng/DON (∼3.4–13.5 μM) after 48 and 72 h (Diesing et al. 2011b). Nevertheless, our finding is supported by a recent study, reporting an underestimation of the anti-proliferative effect of (−)-epigallocatechin-3-gallate (EGCG) via the MTT assay. While ATP- and DNA-based methods revealed anti-proliferative effects of EGCG, the MTT method indicated EGCG-induced proliferation increases of up to 15%. The authors speculate about the ability of EGCG to increase mitochondrial dehydrogenase activity and its potential to reduce MTT. They emphasize that metabolic activity may be influenced by diverse factors, causing considerable variation of results reported from these in vitro assays (Wang et al. 2010).

Only few studies have shown viability data of DON-treated cells via the WST-1 assay. Vejdovszky et al. (2015) reported significant viability decreases after a 24-h treatment of undifferentiated Caco-2 with 10 μM DON. We are the first to evaluate DON toxicity according to the CTG assay in differentiated IPEC-J2. Only Awad et al. (2012) reported significant ATP concentration reduction after 48 h in undifferentiated IPEC-J2 treated with 2.5–10 μM DON.

Based on the excessive viability signals detected by the WST-1, MTT and CTG assays and due to the accumulation of reports, connecting primary steps of apoptosis to mitochondrial alterations (Cossarizza et al. 1994; Petit et al. 1995), we hypothesized that DON may trigger mitochondrial swelling and possibly formation of megamitochondria. The latter was previously reported in HT-29 cells, treated with DON (∼1.7 μM) for 24 h (Krishnaswamy et al. 2010). However, we are the first to examine potential DON-induced mitochondrial swelling or megamitochondria formation in porcine IECs. The loss of the typical thin filamentous mitochondrial network in DON-treated IPEC-J2 and the formation of enlarged and granular-shaped mitochondria located in close vicinity of the nucleus are in accordance with characteristics of mitochondrial enlargement reported in literature. While normal mitochondria are described as thin filamentous structures (“spaghetti”), megamitochondria are granular, round in shape and densely packed around the nucleus (“meatballs”) (Ahmad et al. 2001; Karbowski et al. 1999a).

We hypothesize that the high metabolic activity and ATP production of DON-treated cells, as shown by the MTT, WST-1 and CTG assays, could—at least partially—be due to interferences of DON with mitochondrial morphology. Mitochondrial volume homeostasis ensures structural integrity of the organelle, and increased mitochondrial matrix volume activates the respiratory chain, increasing ATP production and respiration rates (Halestrap 1987, 1989; Lim et al. 2002). It could be hypothesized that while the cell is under energetic stress, where the need for ATP is high, an increase in matrix volume could lead to further activation of the respiratory chain (Kaasik et al. 2007). In fact, a study related to the electron transport chain activity in megamitochondria has revealed a 50% increase in the activity of succinate dehydrogenase activity (Wagner and Rafael 1975). It has also been suggested that disruption of protein synthesis could be a secondary factor contributing to mitochondrial enlargement (Karbowski et al. 1997, 1999a). Consequently, DON-induced ribotoxic stress could be a factor leading to mitochondrial enlargement. Although increases in ROS have been shown to precede formation of megamitochondria, we could not detect DON-induced oxidative stress and therefore cannot support this hypothesis (Karbowski et al. 1999a).

Aside from the comparative analysis of DON, the present study is particularly relevant with respect to DON metabolite, DOM-1. The toxicological relevance of DOM-1, which has been poorly documented, is justified by natural exposure as well as through the frequent use of DON- to DOM-1-converting feed additives. The amount of DON metabolites has not been considered in the regulatory limits fixed by food agencies, due to the lack of data regarding their absorption and toxicity (Maresca 2013). The present study included a thorough cell-based investigation of DOM-1, which has not yet been performed in this manner. Only few studies are available regarding DOM-1 in vitro cytotoxicity. Danicke et al. (2010) assessed DOM-1 cytotoxicity in PBMCs and differentiated IPEC-1 and IPEC-J2 over 48 h, however only via a single MTT assay, and concluded lack of DOM-1 cytotoxicity under these conditions. Sundstol Eriksen et al. (2004) tested the effect of DOM-1 on 3T3 fibroblasts via BrdU incorporation and reported the IC50 of DOM-1 to be ∼55 times higher than that of DON. Nasri et al. (2006) assessed the effect of DOM-1 in Jurkat T cells via a single alamarBlue assay as well as the potential of DOM-1 to induce apoptosis or necrosis via Annexin V staining. Finally, Pierron et al. (2016) recently demonstrated that 30 μM DOM-1 does not affect viability of proliferating Caco-2 cells via the CTG assay. The authors demonstrated that 10 μM DOM-1 does not activate MAPK proteins p38 and Sapk/JNK and showed that bacterial de-epoxidation of DON alters its interaction with the ribosome. Accordingly, both DON and DOM-1 fit into the pockets of the A-site of the ribosomal peptidyl transferase center. However, while DON forms three hydrogen bonds, DOM-1 forms only two, thereby losing its ability to activate MAPK signalling. Thus, our study is not only the first to evaluate and exclude DOM-1 toxicity according to six cytotoxicity assays in parallel, it is also the first to demonstrate a lack of DOM-1-mediated apoptosis via cleaved caspase-3 activation, MAPK signalling or oxidative stress in differentiated porcine IECs. Furthermore, we are the first to test DOM-1 up to a concentration as high as 100 μM. We thereby extend current knowledge regarding the safety of feed additives containing DON- to DOM-1-transforming bacteria.

Considering the high DON susceptibility of pigs, the non-tumorigenic and non-transformed IPEC-J2 is a highly suitable in vitro model (Berschneider 1989; Nossol et al. 2015) to study the impact of DON and DOM-1. While differentiated IPEC-J2 are frequently cultured in two-compartment transwell membrane systems, various investigations have been conducted with differentiated IPEC-1 and IPEC-J2 in conventional well plates (Broekaert et al. 2016; Diesing et al. 2011b; Vandenbroucke et al. 2011; Wan et al. 2013). By culturing IPEC-J2 in 96-well plates for 1 or 21 days, Vandenbroucke et al. (2011) established that, compared to proliferating cells, differentiated porcine IECs show a 10-fold higher DON sensitivity. Using IPEC-1 and IPEC-J2, both cultured in conventional one-compartment plates, Diesing et al. (2011b) monitored expression of tight junction protein zona occludens 1 (ZO-1) and enzyme activity of alkaline phosphatase, a marker of differentiation. By day 4, immunofluorescence staining of ZO-1 revealed a continuous lining around adjacent cells, which remained unchanged until day 21. Alkaline phosphate enzyme activity was remarkably increased between days 2 and 3, remaining stable until day 22. Additionally, the authors demonstrated that in transwell membrane inserts, cells exhibited a similar kinetic as in normal plates, reaching transepithelial electrical resistance (TEER) values above 1 kOhm × cm^2^ after 4 days. Nevertheless, it should be stated that the differentiation of IPEC-J2 in two-compartment cultivation systems has been shown to promote optimal development of a polarized epithelial barrier with differentiated apical and basolateral features, thereby closely mimicking in vivo conditions (Kluess et al. 2015; Nossol et al. 2010). Furthermore, Diesing et al. (2011a) and Kluess et al. (2015) demonstrated that IPEC-J2 react differently to DON, depending on whether the toxin is applied apically or basolaterally in transwell membrane inserts.

Therefore, besides novel aspects with respect to DON effects on mitochondria, we present the first comparative in vitro analysis of DON and DOM-1, based on six different parameters of cytotoxicity. Although frequently neglected in literature, assays based on different cellular parameters reflect differential responses of cellular functions and compartments. Multi-parameter approaches, as presented in this study, permit a reliable in vitro picture of the toxicological relevance of not just DON or DOM-1, but of any given compound, thereby strengthening in vitro tests as a suitable alternative or prerequisite to in vivo animal testing. The study highlights the need for meticulous evaluation of the method used for in vitro assessment of cell viability to improve probabilities to detect toxicants, which might otherwise be underestimated or overestimated.
